# Diet and Fertility Status: Relevance in Health and Disease

**DOI:** 10.3390/nu15071669

**Published:** 2023-03-29

**Authors:** Sara Della Torre

**Affiliations:** Department of Pharmaceutical Sciences, University of Milan, 20133 Milan, Italy; sara.dellatorre@unimi.it

The prevalence of obesity and other metabolic disorders is increasing worldwide [1], leading to detrimental effects on human health, including reproductive performance, and thus affecting the quality of life of a large portion of society [2,3]. Increasing evidence suggests that the fertility status of males and females is linked to several metabolic and inflammatory diseases, in addition to dietary habits.

Throughout evolution, mechanisms have developed to sense nutrients, store energy in case of food abundance and adapt physiological responses to nutrient availability, preventing reproduction in nutrient-poor environments; this strict interplay reached its highest degree of complexity in female mammals [4]. Females are responsible for the energy costs of reproduction, and thus regulatory mechanisms which modulate the interplay with metabolic homeostasis have been positively selected and further perfected during evolution to guarantee procreation only in the presence of favorable energy conditions and to tune the overall metabolism to the demanding energy needs of reproduction [4,5].

In the current obesogenic world, this evolutionary strategy has negative effects on overall health and is also impacting fertility, especially in women, who thus lose the metabolic advantages over men guaranteed by female hormones [4,5,6]. In this view, nutrients cannot be merely considered a source of energy, as they exert a bioactive role by acting on several signaling pathways [7].

As summarized in the review by Jurczewska et al. [8], ovulation-dependent female fertility is influenced by several diet-related factors, especially the excessive intake of high-glycemic-index carbohydrates, large amounts of animal protein, saturated fatty acids, and *trans* fatty acids. It is known that high-fat diet (HFD) regimens negatively impact on several reproductive processes, such as folliculogenesis, oogenesis, and embryo development/implantation, leading to female infertility and transgenerational disorders [9]. Although our currently fragmentary knowledge limits the full comprehension of the underlying mechanisms, current evidence suggests that the negative impact of HFDs on fertility may be due to a direct action on reproductive somatic and germinal cells and/or to an indirect effect mediated by endocrine, metabolic, and immune signaling [9]. The burden of chronic inflammatory diseases, including Crohn’s disease, negatively impact on female reproduction, potentially contributing to irregular menstrual cyclicity, implantation failure, and other negative fertility outcomes [10,11]. 

In women, subfertility and low success of assisted reproductive technology (ART) have been also associated with low trace elements of copper, selenium, and zinc [12], showing that the proper dietary intake of these elements is essential for reproductive health. 

In men, over-exposure to heavy metals such as Cadmium (Cd), which can also occur through the consumption of contaminated water and food, induces structural and functional testicular damage due to oxidative stress, inflammation, and apoptosis [13].

Among the several risk factors for infertility [14], gestational exposure to endocrine disruptors, such as bisphenol A (BPA), in combination with HFD may lead to impaired spermatogenesis in F1 and F2 offspring [15], supporting evidence on the transgenerational inheritance of reproductive disorders as well as of metabolic diseases [16,17,18]. With this in mind, studies aiming to unravel the long-term effects of maternal nutrition [19] may help to further elucidate the relevance and the mechanisms through which gestational nutrients can program metabolic and reproductive functions later in life, as well as in offspring. 

Dietary interventions might represent the most promising and invaluable strategy to limit or prevent metabolic and inflammatory complications associated with, or consequent to, subfertility [10,11,20]. Given the adverse effects of inflammation on reproductive processes, anti-inflammatory diets rich in monounsaturated (MUFA) and ω-3 polyunsaturated (PUFA) fatty acids and flavonoids, and poor in red and processed meat, such as the Mediterranean diet, may improve fertility and ART success in women [10,11]. In insulin-resistant PCOS (polycystic ovary syndrome) adolescents with obesity, lifestyle changes and appropriate early dietary interventions aiming to reduce insulin resistance (i.e., diets rich in carbohydrate with low glycemic index, plant protein, MUFA and PUFA, folic acid, vitamin D, antioxidants, and iron) are recommended approaches to restore ovulation and to protect fertility [20]. 

In males, moderate calorie restriction may have the potential to improve the metabolic profile (i.e., plasma glycemia, glucose-regulating hormones) and reproductive parameters (i.e., plasma testosterone levels, sperm quality, male sexual behavior) [21]. Dietary strategies such as the intake of nutraceuticals, particularly abundant in the Mediterranean diet, can be recommended against Cd-induced testicular injury [13].

Besides fertility, environmentally induced abnormalities in the reproductive cycle should be considered as an important risk factor for female metabolic health. Accordingly, women with impaired or null reproductive cycles (i.e., PCOS women) experience increased incidence of metabolic/inflammatory pathologies, such as non-alcoholic fatty liver disease (NAFLD), metabolic syndrome (MetS), and cardiovascular diseases (CVDs) [4,22]. Nevertheless, more than fertility status per se, the physiological, rhythmic changes of estrogen levels during the reproductive cycle are essential to program and to preserve metabolic homeostasis in females [23,24]. Notably, the lack of the receptor that mediates estrogen action in the liver (hepatic estrogen receptor alpha, ERα) increases female vulnerability to HFD-induced metabolic alterations [25] and an impaired regulation of hepatokines is involved [26]. An alteration of hormonal signaling can have detrimental effects, especially in the female liver, where changes in the transcriptional activity of ERα during the fertile cycle enable the regulation of lipid metabolism according to the energy needs of each reproductive phase [23,24], contributing to sex differences in the regulation of hepatic metabolism [5,27] and in the susceptibility to liver diseases [22,25,28]. The disruption of this hepato–ovarian axis in ovariectomized females leads, indeed, to altered hepatic metabolism [23], which can be, at least in part, rescued by dietary amino acids [29], further demonstrating the potential of nutritional approaches in improving metabolic health when compromised by altered fertility status.

Overall, this Special Issue provides compelling evidence of the relevance of the nutritional status on the mutual relationship between fertility and metabolic homeostasis, as well as between subfertility and metabolic disorders both in males and females. More studies are required to unravel the underlying mechanisms of action and explore the potential of sex-specific nutritional strategies to preserve metabolic homeostasis and fertility in both sexes.
